# Intraoperative Neurophysiological Monitoring during Surgical Correction of Scoliosis for Postoperative Recovery of the Patient’s Motor Function

**DOI:** 10.17691/stm2021.13.5.07

**Published:** 2021-10-29

**Authors:** Yu.S. Arestova, M.S. Sayfutdinov, D.M. Savin, M.Z. Nasyrov, T.V. Ryabykh, S.O. Ryabykh

**Affiliations:** Specialist, Clinic for Spine Pathology and Rare Diseases National Ilizarov Medical Research Center for Traumatology and Orthopedics, Ministry of Health of the Russian Federation, 6 M. Ulyanova St., Kurgan, 640014, Russia; Leading Researcher National Ilizarov Medical Research Center for Traumatology and Orthopedics, Ministry of Health of the Russian Federation, 6 M. Ulyanova St., Kurgan, 640014, Russia; Head of Traumatology and Orthopedics Department No.9 National Ilizarov Medical Research Center for Traumatology and Orthopedics, Ministry of Health of the Russian Federation, 6 M. Ulyanova St., Kurgan, 640014, Russia; Head of the Department of Rehabilitation National Ilizarov Medical Research Center for Traumatology and Orthopedics, Ministry of Health of the Russian Federation, 6 M. Ulyanova St., Kurgan, 640014, Russia; Doctor of Functional Diagnostics National Ilizarov Medical Research Center for Traumatology and Orthopedics, Ministry of Health of the Russian Federation, 6 M. Ulyanova St., Kurgan, 640014, Russia; Deputy Director National Ilizarov Medical Research Center for Traumatology and Orthopedics, Ministry of Health of the Russian Federation, 6 M. Ulyanova St., Kurgan, 640014, Russia

**Keywords:** spinal deformity, scoliosis, electromyography, neuromonitoring, motor-evoked potentials, physiotherapy

## Abstract

**The aim of the investigation** was to study the effect of adverse intraoperative events on the subclinical decrease in the functional state of the sensorimotor system of patients with scoliosis and their early postoperative rehabilitation.

**Materials and Methods:**

The results of the examination of 30 adolescents of 13–16 years old with scoliosis before and after surgical correction were compared. Intraoperative neurophysiological monitoring was used by the method of transcranial evoked motor potentials. The patients were divided into two groups depending on the presence or absence of neurophysiological signs of damage to nerve structures during the operation.

**Results:**

The amplitude of the M-responses of the muscles of the lower limbs in the postoperative period remains at a level close to the initial one, with a noticeable decrease in the amplitude of voluntary electromyography, which is expressed unevenly and to a greater extent in patients with intraoperative signs of hazard for the motor pathways of the spinal cord.

**Conclusion:**

Adverse intraoperative events cause significant changes in the state of the motor system of patients with scoliosis and reduce the effectiveness of rehabilitation treatment in the postoperative period.

## Introduction

Currently, the surgical treatment of severe spinal deformities is symptomatic. It is resorted to when conservative management turns out to be ineffective, unable to cope with worsening the problem. To get insight into the causes of the onset and progression of this group of orthopedic diseases, studies of the structural and functional state of the sensorimotor system of patients are carried out using instrumental methods [1, 2]. The information obtained that way can also be used to optimize postoperative rehabilitation and further conservative treatment [3].

In the surgical treatment of scoliosis, there is a risk of a new neurological deficit in the postoperative period [4, 5]. For the solution of this problem, a toolkit of methods for intraoperative neurophysiological monitoring of the functional state of the spinal cord, called intraoperative neuromonitoring, has been formed [6]. This toolkit includes a method of transcranial evoked motor potentials as a tool for testing the motor system [7, 8]. The situations with critical changes in motor-evoked potentials (MEP) in the absence of postoperative neurological disorders are of interest. Some authors interpret these alterations as false positive responses of the pyramidal tract of the spinal cord [9], however, there is no unanimous opinion on this issue [7, 10]. The results of our studies have shown that even in the absence of postoperative neurological deficit, adverse intraoperative events accompanied by negative changes in the MEP lead to a decrease in neurophysiological sensorimotor parameters [11, 12].

**The aim of the investigation** was to study the effect of adverse intraoperative events on a subclinical decrease in the functional state of the sensorimotor system of patients with scoliosis and their early postoperative rehabilitation.

## Materials and Methods

The study included 30 adolescents (11 males and 19 females) aged 13–16 years (average age of 14.50±0.16 years) with scoliosis of different ethology: idiopathic scoliosis (n=16); congenital deformity of the spine (n=5); spinal deformity associated with systemic diseases of the skeleton (n=3); neurogenic scoliosis (n=6). Deformity localization: thoracolumbar (n=25); chest (n=3); cervicothoracolumbar (n=2). The deformation angle varied from 40 to 115°. The parameters of the patients’ physical development were assessed taking into account their growth data [13].

Motor (M) responses of the muscles of the lower limbs were obtained with monopolar cutaneous abduction and supramaximal stimulation of the corresponding nerves, electromyograms were made at voluntary maximal tension (cutaneous bipolar muscle abduction). The cerebrospinal index (CSI) proposed by Shein was calculated [14]. M. rectus femoris, m. tibialis anterior, m. gastrocnemius lateralis, m. extensor digitorum brevis, m. flexor digitorum brevis of the left and right lower limbs were used as muscles-indicators. The studies were carried out before surgical correction of the spinal deformity and two weeks after the intervention. The obtained findings were compared with the results of the examination of practically healthy volunteers [15]. Intraoperative neurophysiological monitoring was carried out by the method of transcranial-evoked motor potentials.

The patients were divided into two comparison groups: group 1 (n=20) included patients after a smooth operation, who did not show any neurophysiological signs of hazardous changes in the state of the spinal cord; the patients of group 2 (n=10) demonstrated a significant inhibition of the MEP up to their complete disappearance during the operation, which was indicated by a significant decrease in the functional state of the pyramidal system of the spinal cord. In the postoperative period, these patients showed no clinical signs of neurological disorders.

A course of physiotherapy which included physical exercises aimed at respiratory and general strengthening was prescribed to all the subjects in the postoperative period.

The study was conducted in accordance with the ethical standards set out in the Declaration of Helsinki (2013) and was approved by the Ethics Committee of the National Ilizarov Medical Research Center for Traumatology and Orthopedics (Kurgan, Russia). Informed voluntary consent was obtained from all the patients over 15 years old and the parents of the patients under 15 years old, in accordance with the Federal Law of the Russian Federation “On the fundamentals of protection of the public health” (2011).

**Statistical analysis.** Mathematical data processing was performed using the Microsoft Excel 2010 software package and the Attestat package integrated with it, as well as the IBM SPSS Statistics 21.0 software package. The Kolmogorov–Smirnov method was used to assess the nature of the statistical distribution of the analyzed parameters of electromyography (EMG). The mean values of the amplitude of the evoked and voluntary EMG and the standard error of the mean were calculated. The data were presented as M±m. The coefficient of variation (CV) was used as a measure of variability, that is the ratio of the standard deviation to the mean expressed as a percentage. The significance of differences in the groups due to the normal nature of the statistical distribution was determined using the t-test for independent and pairwise conjugate samples with a preliminary assessment of the condition for equality of variances using the Levene’s test. Differences were considered statistically significant at p<0.05.

## Results

The parameters of physical development, taking into account the patients’ growth, were distributed in the centile corridors for SD2neg, SD1neg, SD0, i.e. with a prevalence of underweight in 21 patients (70%). Lower body weight was found in all the patients with congenital (SD1neg in 5 patients), systemic (SD2neg in 2 patients, SD1neg in 1 patient), and neurogenic (SD2neg in 2 patients, SD1neg in 4 patients) spinal deformity and in 7 out of 16 patients with idiopathic scoliosis (SD1neg).

The results of neurophysiological testing of the functional state of the motor system revealed that the average amplitude of the M-response of the muscles of the lower limbs in the comparison groups did not differ significantly (p>0.05). For 3 out of 5 tested muscles, it decreased, regarding the norm, by 6.5–31.2% in group 1 and by 9.3–27.5% in group 2. A decrease in the M-response of m. extensor digitorum brevis in both groups was statistically significant (p<0.05) (Table 1).

**Table 1:** Mean values of the amplitude of the M-response of the muscles of the lower limbs, M±m

Muscle	Before surgery			After surgery			Norm (mV)
	n	M-response amplitude (mV)	CV (%)	n	M-response amplitude (mV)	CV (%)	
***Group 1***							
m. rectus femoris	36	19.7±0.6	17.0	34	18.1±0.7	22.0	21.1±0.4
m. tibialis anterior	40	8.3±0.4	30.4	38	9.3±0.4	27.4	7.9±0.2
m. extensor digitorum brevis	40	7.3±0.5*	47.3	38	6.9±0.6*	54.4	10.6±0.4
m. gastrocnemius lateralis	38	29.3±1.6	34.8	38	30.1±1.6	32.2	31.5±0.5
m. flexor digitorum brevis	38	19.0±0.9	30.6	38	20.3±0.9	27.3	18.1±0.7
***Group 2***							
m. rectus femoris	18	19.1±0.8	17.0	15	17.2±0.7	16.9	21.1±0.4
m. tibialis anterior	20	8.4±0.4	20.2	20	8.6±0.6	31.4	7.9±0.2
m. extensor digitorum brevis	16	7.7±0.8*	39.9	16	6.7±0.9*	55.0	10.6±0.4
m. gastrocnemius lateralis	20	26.6±1.8	29.5	20	26.5±1.7	28.9	31.5±0.5
m. flexor digitorum brevis	18	18.6±1.3	30.7	18	19.7±1.5	31.8	18.1±0.7

Note: * statistically significant decrease in the M-response amplitude in comparison with the norm (p<0.05); n is the number of tested muscles.

The mean-sample values of EMG parameters at the maximum voluntary muscle tension of the lower limbs (Table 2) in the comparison groups did not differ statistically significantly (p>0.05), with the exception of m. rectus femoris, for which the mean amplitude in the preoperative period was statistically significantly higher (p<0.05) in the patients of group 2 compared with group 1. Moreover, in all the cases, the amplitude was significantly reduced (p<0.05) relative to the norm by 12.5–44.1% in group 1 and by 31.3–34.3% in group 2, with the exception of the values for m. rectus femoris in the group 2. The variability of the amplitude of voluntary EMG is comparable to the variability of the M-responses.

**Table 2:** Mean values of electromyography parameters at maximal voluntary muscle tension of the lower limbs, M±m

Muscle		Before surgery			After surgery			Norm (mV)
		n	EMG parameters	CV (%)	n	EMG parameters	CV (%)	
***Group 1***								
m. rectus femoris	A	36	0.36±0.03*	45.0	37	0.30±0.03*	61.7	0.41±0.04
	f	26	293±17	29.2	28	271±12	22.9	238±18
m. tibialis anterior	A	39	0.42±0.03*	40.5	37	0.45±0.03*	42.3	304±14
	f	26	436±13	14.8	30	428±25	32.4	0.65±0.03
m. gastrocnemius lateralis	A	35	0.22±0.02*	42.8	34	0.21±0.02*	45.8	0.40±0.05
	f	26	404±21	26.5	30	347±16	24.7	268±22
***Group 2***								
m. rectus femoris	A	18	0.46±0.04^v^	39.7	17	0.27±0.03*^+^	47.8	0.41±0.04
	f	12	349±20	20.2	12	282±29	36.2	238±18
m. tibialis anterior	A	19	0.45±0.06*	56.0	15	0.38±0.03*	34.6	0.65±0.03
	f	12	408±32	27.0	12	386±32	28.5	304±14
m. gastrocnemius lateralis	A	18	0.26±0.03*	50.0	16	0.24±0.03*	48.4	0.40±0.05
	f	12	413±18	15.0	12	363±25	23.5	268±22

Note: * statistically significant difference in values compared to the norm, ^v^ with group 1, ^+^ with the preoperative level; p<0.05; n is the number of tested muscles; A is the amplitude (mV); f is the number of turns per second.

According to the character of summation in action potentials of motor units, all the registered patterns of voluntary EMG can be divided into two types: saturated EMG and reduced EMG. In the first case, the electrogram of voluntary activity presents a broken line of a complex type that repeatedly crosses the zero level in random order. The entire segment of the recording is filled evenly with spikes of electrical activity converting into each other. Only saturated patterns were used to calculate the mean frequency of voluntary EMG. Reduced EMG is an alternation of saturated spikes of activity with periods of electrical silence during the epoch of analysis. In the patients of group 1, the reduced EMG patterns were registered bilaterally and accounted for 31.5–35.0% of all observations. In group 2, reduced EMG occurred in 40.0% of the cases for all the muscles.

During surgery, cases of a decrease in the MEP amplitude by more than 50% up to complete disappearance were recorded by the method of MEP testing in all the patients of group 2. At the same time, the surgeon and anesthesiologist took action to eliminate the problem (changing the position, up to removal of the structural elements, tension of corrective forces, injecting glucocorticoids). The critical changes in the MEP were reversible (lasting no more than 15 min with a subsequent return to the initial level) and irreversible (when the MEP amplitude was not restored until the end of the operation). However, all the patients in the postoperative period had no clinical signs of sensorimotor disorders.

After surgical correction of the spinal deformity, the amplitude of M-responses did not change significantly (p>0.05) in both groups (see Table 1). Table 2 demonstrates that the patients with a smooth course of surgery (group 1) had an insignificant (p>0.05) decrease by 6.3–16.2% in the EMG amplitude relative to the initial level in the postoperative period. In group 2, this decrease was more pronounced and amounted to 9.6–41.7% compared to the preoperative level. It is minimal for the calf muscles (p>0.05) and maximal for the rectus femoris muscle (p<0.05). The decrease in the EMG amplitude for the tibialis anterior muscle had intermediate values but remained statistically insignificant (p>0.05). At the same time, the number of patterns with reduced voluntary EMG in group 1 decreased after surgery by 5–10% (up to 20–30%), while in group 2 it remained practically unchanged, it accounted for 40%, with the exception of m. rectus femoris on the right, where the number of patterns decreased by 6%.

## Discussion

The obtained results indicate that the functional deterioration of the neuromuscular system in adolescents with scoliotic deformity of the spine is mainly due not to structural, but functional changes, since the M-responses of most muscles differ insignificantly from the norm, and the voluntary amplitude of EMG is significantly reduced. The decrease in the level of voluntary activity can be explained by the restructuring of the central mechanisms of motor activity control under altered biomechanical conditions and against the background of impaired interaction of the central nervous system with the peripheral part of the effector apparatus [16].

This statement can be confirmed by the cerebrospinal indices of the patients (Table 3). When comparing the baseline EMG characteristics of adolescents from the groups 1 and 2, no significant differences were revealed, i.e. the occurrence of intraoperative negative events is not predetermined by the state of the patient’s neuromuscular system.

**Table 3:** Mean values of the cerebrospinal index (CSI) of the muscles of the lower limbs, M±m

Muscles	Group	Before surgery		After surgery		Norm (%)
		n	CSI (%)	n	CSI (%)	
m. rectus femoris	1	38	1.8±0.1	34	1.6±0.1	
	2	18	2.1±0.3	15	1.7±0.2	3.2–3.7
m. tibialis anterior	1	39	5.4±0.3	37	4.6±0.3	
	2	20	5.3±0.8	20	3.9±0.6	8.9–9.2
m. gastrocnemius lateralis	1	37	0.8±0.1	35	0.8±0.1	
	2	18	0.9±0.1	18	0.8±0.1	1.5–1.6

Notе: n is the number of tested muscles.

Surgical correction of spinal deformity provides a powerful multifactorial effect on the body [17, 18]. In particular, reflex defense mechanisms limiting the motor activity of the patients in the postoperative period are activated under the influence of changes in the balance of specific and nonspecific components of somatosensory afferentation, as well as due to the intense flow of sensory impulses from tissue interoceptors (including nociceptors) [19–21]. Initially, these protective mechanisms allow the body to optimally distribute internal resources during the recovery period, but then, due to the tonic nature of protective reflexes, they become an obstacle in overcoming postoperative hypodynamia and hypokinesia. Postoperative physiotherapy with therapeutic exercise [22, 23] and electrical stimulation [24, 25] contribute to a smooth weakening of protective inhibition.

An almost complete return of the functional state of the neuromuscular apparatus to the initial level after two weeks of rehabilitation exercise was found in the patients with a smooth course of surgical intervention (group 1), while in group 2, the recovery process was noticeably slower despite the absence of visible clinical problems. The essential role of protective reflex mechanisms limiting motor activity was manifested in intergroup differences in the dynamics of reduced patterns of voluntary EMG. Reduced EMG patterns arise due to an increased activity of the inhibitory systems, limiting the intensity of the spinal motoneuronal pools of the corresponding muscles [26]. In group 1, there was a marked decrease in the number of reduced EMG patterns, which indicates a weakening of the inhibitory effects on the motor system. In group 2, this parameter practically did not change, i.e. the given complex of physiotherapy exercises was not sufficient to overcome the inhibitory effects of the surgical intervention. Thereby, it is obvious that negative intraoperative events contribute to the stabilization of protective inhibition mechanisms in the postoperative period.

When patients do not show clinical signs of motor disorders in the postoperative period, no special physiotherapy exercise complexes or complementary physiotherapy, for example, myoelectrostimulation, are indicated for them [27–29]. However, in the absence of clinical signs of neurological disorders, instrumental research methods make it possible to identify more pronounced deviations of EMG parameters in the patients who have undergone negative intraoperative events, than in those after a smooth course of surgery. [11, 12]. This condition has been defined as subclinical sensorimotor deficit [30].

A slight decrease in the amplitude of voluntary EMG relative to the initial level, observed at the follow-up examination, suggests that the physiotherapy exercise complex used in the postoperative period helps to restore the functional state of the muscles of the lower limbs to a level close to the initial one. However, although unfavorable intraoperative events (changes in the state of the motor system) do not lead to clinically pronounced disorders, they significantly affect the nature of the motor activity of patients in the postoperative period, which reduces the effectiveness of the use of remedial physiotherapy.

## Conclusion

Adverse intraoperative events cause changes in the motor system state, which significantly affect the nature of the motor activity of the patients in the postoperative period. This reduces the effectiveness of restorative therapy and may affect the treatment of spinal deformity.

Further studies are needed to substantiate the criteria for evaluating the effectiveness of rehabilitation treatment in order to revise the grounds for prescribing rehabilitation measures, and, in particular, for the development of specialized physiotherapy exercise complexes and complementary therapy (myoelectrostimulation) in the presence of adverse intraoperative events.
